# Supplementary material for: KCTD15 Protein Expression in Peripheral Blood and Acute Myeloid Leukemia
Source: Diagnostics (Basel). 2020 Jun 4;10(6):371. doi: 10.3390/diagnostics10060371 (PMC7345863; doi:10.3390/diagnostics10060371)
Supplement: Supplementary file 1 [file diagnostics-10-00371-s001.pdf]

Supplementary Table 1. KCTD15 intensity of expression (mean +/- SD) in circulating lymphocytes, monocytes and granulocytes.

Background		Lymphocytes		Monocytes		Granulocytes	
Mean	SD	Mean	SD	Mean	SD	Mean	SD
4089	±1814	7672	±7574	21157	±3772	46306	±11656
4089	±1814	5728	±3299	23754	±6242	46720	±14884
4089	±1814	8188	±2317	18221	±4095	44471	±11118
4089	±1814	2804	±3540	15730	±5098	37465	±9419
4089	±1814	9001	±3692	17353	±5338	39719	±7172
4089	±1814	6057	±2978	12448	±3710	15588	±5517
4089	±1814	6647	±4407	18097	±6031	49330	±17893
4089	±1814	7419	±2971	15941	±6129	25932	±13305
4089	±1814	7194	±4336	16796	±9522	51458	±20572
4089	±1814	6303	±4544	19205	±6559	37803	±10339
4089	±1814	7670	±3523	15097	±6453	40114	±17322
4089	±1814	8109	±3384	16065	±6486	33762	±10946
4089	±1814	6735	±2420	14062	±5353	35094	±14334
4089	±1814	8897	±3317	17201	±6675	33431	±12748
